# Ileal metastasis of colorectal cancer diagnosed by double-balloon endoscopy and resected via laparoscopy: A case report

**DOI:** 10.1016/j.ijscr.2025.111072

**Published:** 2025-02-19

**Authors:** Masataka Nakagawa, Daisuke Sumitani, Keiso Matubara, Hiroshi Ota, Masatsugu Yano

**Affiliations:** Department of Surgery, Medical Corporation Hiroshima Hospital, 3-1-36 Hutabanosato, Higashi-ku, Hiroshima 732-0057, Japan

**Keywords:** Small bowel metastasis of colorectal cancer, Double-balloon endoscopy

## Abstract

**Introduction:**

Small bowel metastasis of colorectal cancer (CRC) is rare, with a 3.8 % occurrence. Preoperative diagnosis was considered challenging; however, with the development of various endoscopes, diagnosis may now be possible.

Most small bowel metastases of CRC are systemic metastatic events, such as direct invasion or disseminated metastasis. Therefore, R0 surgery is difficult to achieve, and local treatment is infrequent.

**Presentation of case:**

A 70-year-old woman underwent laparoscopic left hemicolectomy for transverse colon cancer in 2022 and her final staging was pT4a, N1b, M0, pStage IIIb. One year after surgery, her carcinoembryonic antigen (CEA) level was elevated, and computed tomography (CT) showed no evidence of neoplastic lesions; however, positron emission tomography (PET) showed a 1 cm nodule with a high SUVmax:9.1 concentration near the uterus, suggesting the possibility of a small bowel tumor. Double-balloon endoscopy (DBE) revealed a submucosal tumor in the ileum. A biopsy could not be performed; however, the lesion was marked with ink dots and clips near the lesion. The lesion was diagnosed as solitary, and the patient underwent laparoscopic partial ileal resection. The tumor was located approximately 60 cm from the end of the ileum on the mesenteric side of the mouth, and it was impossible to determine whether it was an extramural or intraluminal lesion. The patient had a good postoperative course, and histopathologic examination revealed small bowel metastasis of transverse colon cancer, with tumor cells infiltrating from the subserosal layer to the intrinsic muscularis propria. The patient has been under observation for 1 year and 4 months after surgery without recurrence.

**Discussion:**

Small bowel metastases of CRC are very rare and have a poor prognosis; DBE can be used to identify neoplastic lesions in the ileum that could not be determined as extraintestinal or small bowel lesions by CT or PET alone. By marking the lesion with dots of ink and a clip, the lesion was determined to be solitary and amenable to R0 surgery. Laparoscopic surgery was chosen because of the ease of confirming the markings near the lesion and because it was minimally invasive. Furthermore, laparoscopic surgery allowed observation of the subdiaphragm, pelvic floor, and entire abdominal cavity. This report is the only case in which ink dots and clips were employed during DBE and subsequently utilized when laparoscopic surgery was performed.

**Conclusion:**

We report a case involving a single site of small bowel metastasis after CRC surgery in which the patient underwent laparoscopic resection of the small intestine after locating the metastatic site with DBE and was successfully treated without recurrence. We conclude that if R0 surgery is possible for a single site of small bowel metastasis, it may contribute to an improved prognosis. Endoscopy is useful for detecting small intestinal tumors, and a single site of small bowel metastasis is a good indication for laparoscopic resection.

## Highlights

• We successfully detected and resected small bowel metastases of CRC using DBE.
• The use of DBE with marking the lesion with dots of ink and a clip in Small bowel metastases in CRC facilitated tumor identification at the time of surgery.
• Marking the lesion with dots of ink and a clip enabled laparoscopic surgery and easy observation of the subdiaphragm, pelvic floor, and entire abdominal cavity.
• This report is the only case in which ink dots and clips were employed during DBE and subsequently utilized when laparoscopic surgery was performed.

## Abbreviations

CRC: colorectal cancer
CEA: carcinoembryonic antigen
CT: computed tomography
PET: Positron emission tomography
DBE: double-balloon endoscopy
CE: Capsule endoscopy

## 1 Introduction

Small bowel metastasis of CRC is infrequent, occurring in 3.8 % of cases [1]. Most small bowel metastases of CRC are systemic events, such as direct invasion or disseminated metastasis. Preoperative diagnosis is difficult, and there is no established treatment for local recurrence, although some reports suggest that the prognosis for patients who undergo small bowel resection is relatively good [2]. We report a case of recurrence-free progress 1 year and 4 months after surgery for a single site of small bowel metastasis that appeared 1 year after CRC surgery.

This case is reported according to the SCARE 2023 criteria [3].

## 2 Presentation of case

**Fig. 1:**
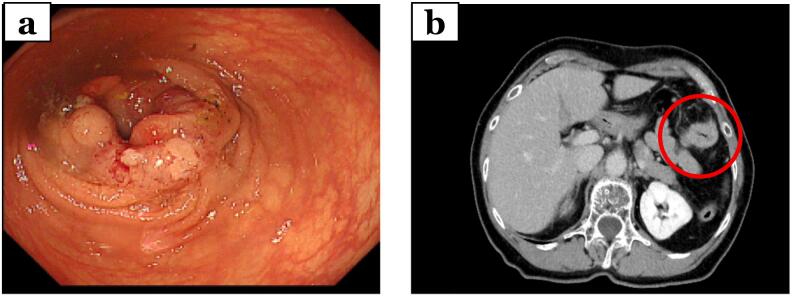
Image findings for diagnosis of transverse colon cancer. (a) Colonoscopy revealed a type 2 tumor in the transverse colon. (b) Computed tomography (CT) revealed a 5 cm mass in the splenic curvature of the transverse colon.

**Fig. 2:**
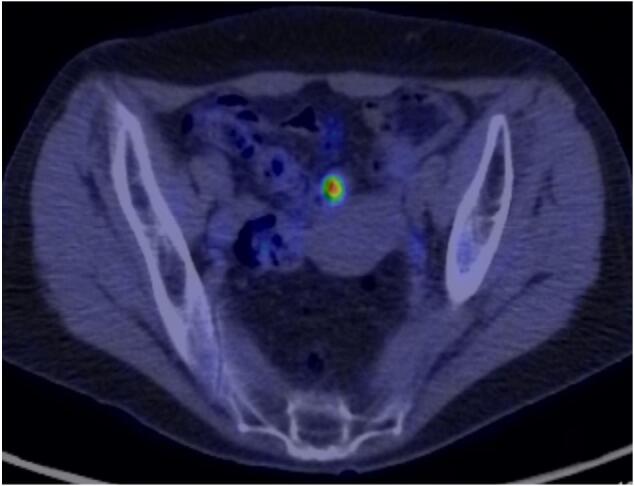
PET-CT findings in the setting of suspected metastatic disease. PET-CT scan showed an accumulation image of SUVmax 9.1 in the small intestine near the uterus.

**Fig. 3:**
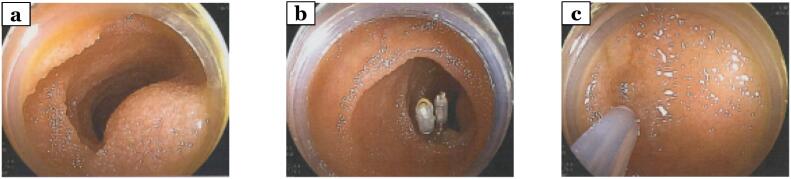
Double-balloon endoscopic (DBE) findings at the time of diagnosis of small bowel metastasis. (a) DBE was used to confirm the presence of neoplastic lesions in the ileum. (b,c) Clipping and point inking were performed in the vicinity of the tumor with DBE.

A 70-year-old woman presented with fecal occult blood. Blood tests revealed abnormally low levels of hemoglobin (10.6 g/dL) and high carcinoembryonic antigen (CEA) levels (55.7 ng/mL). Colonoscopy revealed a type 2 tumor in the transverse colon. Computed tomography (CT) revealed a 5 cm mass in the splenic curvature of the transverse colon (Fig. 1). No other distant metastases were observed. She was diagnosed with CRC (T3 [SS], N0, H0, P0, M0, cStage IIa). Laparoscopic left hemicolectomy was performed, and the local histopathologic results were diagnostic of pT4a(SE), N1b, H0, P0, M0, pStage IIIb. Postoperative adjuvant chemotherapy was not requested, so the patient was followed up. One year postoperatively, blood tests showed a re-elevation of CEA (13.5 ng/mL). CT scan results showed no evidence of neoplastic lesions; however, positron emission tomography-computed tomography (PET-CT) scan results showed an accumulation image of SUVmax 9.1 in the small intestine near the uterus (Fig. 2). A neoplastic lesion was suspected. Capsule endoscopy (CE) was performed, and an isolated lesion was found in the ileum. Double balloon endoscopy (DBE) was performed. Biopsy was challenging due to difficulty in manipulating the forceps, but a confirmation of the presence of a neoplastic lesion in the ileum and dot marking in the vicinity of the lesion were carried out. The vicinity of the lesion was marked with a dot of ink and a clip (Fig. 3). A laparoscopic partial ileal resection was conducted because the tumor was solitary. The patient progressed uneventfully after surgery and was discharged 6 days postoperatively. The tumor was located 60 cm from the end of the ileum on the mesenteric side of the mouth. It could not be determined whether it was an extramural or intramural lesion. Histopathologic examination of the resected specimen revealed proliferation of moderately differentiated ductal adenocarcinoma cells from the subserosal layer to the intrinsic muscularis propria. This led to a diagnosis of small bowel metastasis of CRC. It has now been 2 years and 7 months since the original hemicolectomy and there has been no recurrence. Postoperatively, adjuvant chemotherapy was suggested, but was not performed because the patient did not want it.

## 3 Discussion

Small bowel tumors represent only 2 % of all gastrointestinal neoplasms and comprise primary tumors such as adenocarcinomas, gastrointestinal stromal tumors, carcinoids, and lymphomas [3]. The most common metastatic sites of CRC are the liver, lung, local sites, and other locations, in that order. Small bowel metastasis is included in the “other locations” category (3.8 %) and exhibits an infrequent recurrence [1].

Small bowel metastasis of CRC is very rare, with only eleven cases reported in the literature, including the present report (Table 1). There are three types of metastases. Tumor embolization in the submucosa or muscularis propria of the intestinal tract, either hematogenous or lymphogenous, can lead to metastases (vascular metastasis), or a tumor that has disseminated or infiltrated the serosa or mesentery may develop continuously in the intestinal wall (peritoneal dissemination), while tumor cells that have been released or shed into the intestinal tract may implant and develop in the intestinal mucosa (via the intestinal lumen) [5].

**Table 1:** Reported cases of small bowel metastases from colorectal cancer.

Author / year	Cases	Age/gender	Elevated tumor markers	Symptoms (n)	Primary location (n)	Detected by DBE	Dot marking	Open/LS surgery	Metastatic location	Duration from initial surgery
Bailey et al. / 2006 [11]	1	NA	NA	Bleeding	NA	Yes	No	Open	NA	NA
Spada et al. / 2008 [12]	3	67/M 45/M 72/F	NA	Bleeding Bleeding Bleeding	NA NA NA	Yes	No	Open	Ileum Ileum Ileum	NA NA NA
Rondomonti et al. / 2008 [13]	2	56/M NA	NA	Bleeding NA	NA NA	Yes	No	Open	Ileum NA	1 months NA
Huerta et al. / 2009 [14]	1	NA	NA	Obstruction	NA	None	No	NA	NA	>3 years
Kojima et al. / 2010 [10]	7	NA	Yes	Obstruction (4) Melena (1) Fatigue (1) none (1)	R colon T colon L colon Rectum	No	No	Open	Jejunum (5) Ileum (2)	Synchronous (2) Mean, 43 months (5)
Thoma et al. / 2011 [15]	3	73/F 56/M 73/M	NA	Anemia Bleeding Bleeding	R colon Rectum L colon	Yes	No	Open	Ileum Ileum Jejunum	NA NA NA
Iwamuro et al. / 2015 [16]	1	57/M	NA	NA	S colon	Yes	No	NA	Duodenum	NA
Meshikes et al. / 2016 [17]	1	67/M	NA	Obstruction	L colon	No	No	Open	Jejunum	3 years
Paraskevas et al. / 2017 [9]	1	71/M	NA	None	Rectum	No	No	Open	Ileum	28 months
Uejima et al. / 2020 [18]	1	63/M	Yes	anemia	Rectum	No	No	Open	Jejunum	2 years 9 months
Our case / 2024	1	65/W	Yes	None	T colon	No	Yes	LS	Ileum	14 months

Abbreviations; NA, not available, R colon, right-sided colon, T colon, transverse colon, L colon, left-sided colon, S colon, sigmoid colon, LS, Laparoscopic.

Most small bowel metastasis of CRC occurs via peritoneal dissemination. Patients with a single site of small bowel metastasis such as the present case are very rare. In this case, adenocarcinoma cells were present in the intrinsic muscularis propria and submucosa. No adenocarcinoma cells were found in the serosal layer. Therefore, we considered the possibility of vascular metastasis to be high in this case, if left untreated. The clinical presentation of small bowel metastasis is often atypical. The majority of small bowel metastasis cases that are reported are diagnosed incidentally at the onset of lower gastrointestinal bleeding or bowel obstruction. In the case of the patient described here, small bowel metastasis of CRC was detected by elevated circulating levels of tumor markers and results of a PET-CT scan. Subsequently, CE and DBE were carried out because PET-CT scans could not ascertain whether the lesion was an intra-abdominal lesion near the uterus (extraintestinal lesion). In the past, small bowel metastasis of CRC was often diagnosed by radio enterography or CT, but the recent advent of techniques such as CE and DBE have facilitated the detection of these tumors [6]. CE and DBE have been shown to be superior to CT, barium follow-through, and enteroclysis [6]. However, CE has been reported to have an 18.9 % chance of missing tumor lesions during detection [7]. Further, a biopsy may be difficult in DBE [8]. In fact, the DBE forceps were difficult to manipulate in the present case, making biopsy challenging. Instead, by using DBE, we were able to identify a neoplastic lesion in the ileum that could not be characterized as an extraintestinal or small bowel lesion by CT or PET alone and proceeded to mark it with a dot of ink and a clip. As a result of these intraoperative developments, it was determined that the lesion was solitary and that R0 surgery was feasible. Laparoscopic surgery was also selected because of the ease of identifying the markings near the lesion and the minimally invasive nature of the procedure. Additionally, laparoscopic surgery allowed observation of the subdiaphragmatic and pelvic floor and the entire abdominal cavity. Among such previously reported cases, the present report is the only case in which a dot of ink and a clip were employed during DBE and utilized when laparoscopic surgery was performed subsequently.

The prognosis for small bowel metastasis of CRC is generally poor and whether a simple R0 resection with localized resection is oncologically equivalent to a radical resection remains to be elucidated [9]. Kojima et al. reported recurrence-free survival in six of seven cases of CRC where resection was carried out to treat small bowel metastasis [10]. Similarly, in this report, the patient has been recurrence-free for 2 years and 7 months after resection. This suggests that R0 surgery for a single site of small bowel metastasis, if feasible, may contribute to improved prognosis, although this remains controversial. The choice between open laparotomy and laparoscopy also requires careful consideration. It is generally accepted that open laparotomy facilitates a thorough evaluation of the abdominal cavity. In contrast, laparoscopic surgery is considered minimally invasive and results in a shorter hospital stay [7]. In the present case, CE and DBE were performed preoperatively, and the metastatic site was marked with a dot of ink, while a clip was placed in the vicinity of the tumor to facilitate intraperitoneal retrieval, allowing for a minimally invasive procedure.

## 4 Conclusions

In conclusion, we suggest that if R0 surgery is feasible for a single site of small intestinal metastasis, it may contribute to improved prognosis. Endoscopy is useful for small intestinal tumors, and solitary small bowel metastasis is a good indication for laparoscopic surgery.

## Consent

Written informed consent was obtained from the patient for the publication of the case and any accompanying images. A copy of the written consent is available for review by the Editor-in-Chief on request.

## Ethical approval

The ethics committee of our institution approved all procedures in this case.

## Guarantor

Masataka Nakagawa, Daisuke Sumitani, Keiso Matubara, Hiroshi Ota, and Masatsugu Yano.

## Research registration number

None.

## Funding

No funding.

## Author contribution

MN drafted the manuscript. DS supervised drafting of the manuscript.KM, HO and MY performed the surgical procedure and perioperative management. All the authors have read and approved the final version of the manuscript.

## Conflict of interest statement

The authors declare no conflict of interests for this article.
